# Chronic heat stress compromises egg production and quality parameters through changes in blood biochemistry and uterine gene expression in laying hens raised under cage-free environment

**DOI:** 10.3389/fphys.2026.1770955

**Published:** 2026-01-23

**Authors:** Bikash Aryal, Shuja Majeed, Bikas R. Shah, Nimra Khalid, Lingying Zhao, Lisa Bielke, Qiuhong Wang, Ali Nazmi

**Affiliations:** 1 Department of Animal Sciences, College of Food, Agricultural and Environmental Sciences, The Ohio State University, Wooster, OH, United States; 2 Department of Food, Agricultural, and Biological Engineering, The Ohio State University, Columbus, OH, United States; 3 Prestage Department of Poultry Science, College of Agriculture and Life Sciences, North Carolina State University, Raleigh, NC, United States; 4 Department of Veterinary Preventive Medicine, College of Veterinary Medicine, The Ohio State University, Columbus, OH, United States; 5 Food for Health Discovery Theme, The Ohio State University, Columbus, OH, United States

**Keywords:** blood biochemistry, cage-free, egg production, gene expression, heat stress

## Abstract

Heat stress (HS) possesses a significant threat to poultry production systems, compromising bird health, performance, and profitability. As the egg industry transitions from conventional cage systems to cage-free (CF) systems, understanding the physiological and production impacts of HS is important. This study evaluated the effects of chronic cyclic HS on egg production, egg quality, blood biochemical parameters and shell gland gene expression in commercial laying hens raised in CF housing system. A total of 240 Hy-Line W-36 hens (26 weeks old) were randomly assigned to thermoneutral (TN, 22 °C) or HS conditions (35 °C for 12 h/day, 22 °C for 12 h/day) for 8 weeks. Each treatment included six pens (20 hens/pen; 0.139 m^2^/hen). Body weight, feed intake, and body temperature were measured weekly; egg production and quality were recorded throughout the trial; and blood samples and shell gland tissues were collected at the end of the trial for biochemical and gene expression analyses. Chronic HS significantly (p < 0.05) reduced body weight and feed intake, with HS birds weighing approximately 82 g less per bird and consuming about 27 g less feed/bird/day, however feed conversion ratio remained similar between TN and HS groups. HS caused a reduction in egg production (%) during the first 3 weeks of the experiment, after which the egg production stabilized and became comparable between the TN and HS groups. The HS hens laid significantly (p < 0.001) lighter eggs with weaker shell breaking strength compared with the TN group. Additionally, chronic HS decreased (p < 0.05) blood pH, while increasing partial pressure of carbon dioxide (PCO_2_), and ionized calcium (iCa) levels. The expression of key shell gland genes involved in mineralization, including calbindin 1 (*CALB1*), solute carrier family 4 member 9 (*SLC4A9*), and osteopontin (*OPN*) was downregulated in HS chickens. Collectively, these findings indicate that chronic HS negatively impacted layer performances and eggshell quality in CF housing, likely through disruptions in blood biochemical homeostasis and shell gland gene expression.

## 1 Introduction

As global temperatures continue to rise, heat stress (HS) has become a significant challenge in animal production, particularly in tropical or subtropical regions. A study published over 2 decades ago estimated $128 - $165 million annual economic losses from HS in the poultry industry in the United States, with projections indicating these losses will continue to increase (St-Pierre et al., 2003). The commercial poultry strains selected for rapid growth rates or high egg production are negatively impacted by HS because of their increased metabolic rates, higher heat production, and reduced tolerance to heat (Gogoi et al., 2021; Nawaz et al., 2023). The adverse effects of HS on chickens housed in cage system include slower growth, lower egg production rate and quality, greater disease susceptibility, and higher mortality rates (Tang et al., 2021; Kim et al., 2024). Under the HS condition, chickens exhibit various behavioral and physiological responses, such as panting and wing lifting as means of evaporative cooling (Richards, 1970; Kim et al., 2021), and reduced feed intake as an adaptive strategy to decrease the internal heat generation (Renaudeau et al., 2012; de Souza et al., 2016). However, panting causes hyperventilation and significant carbon dioxide loss, which eventually results in respiratory alkalosis characterized by decreased H^+^, increased HCO_3_^−^, and elevated blood pH (Teeter et al., 1985; Franco-Jimenez et al., 2007). Evidence suggests that acid-base imbalance resulted from HS affects the eggshell quality by decreasing calcium and carbonate secretion in shell gland (Mongin, 1978; Oluwagbenga and Fraley, 2023). It has been demonstrated that respiratory alkalosis developed because of hyperventilation adversely affects the activity of an enzyme carbonic anhydrase, important for eggshell formation (Ahmad and Sarwar, 2006). However, the severity of acid-base disturbance and the importance of maintaining acid-base homeostasis during HS condition in chickens is less appreciated. Moreover, HS in chickens is often accompanied by increased mitochondrial reactive oxygen species (ROS) production, ultimately developing into oxidative stress (Mujahid et al., 2005; Akbarian et al., 2016). Although low levels of ROS are important in various signaling pathways, over production of ROS results in oxidative damage to carbohydrate, proteins, fatty acids and DNA (Habashy et al., 2019; Katerji et al., 2019). In chicken ovary, oxidative stress was shown to promote follicular cell apoptosis by downregulating genes involved in cell survival and hormone synthesis while upregulating the pro-apoptotic factors (Wang et al., 2021).

To improve layer chicken welfare, the U.S. egg industry targets to converting 66% of its national hen inventory from conventional cage (CC) housing to cage-free (CF) housing by 2026 (UEP, 2024). However, studies have reported that the current CF system is 36% more expensive (Matthews and Sumner, 2015) and 7–8 times dustier (Zhao et al., 2015), emits higher levels of ammonia, and enriches more pathogens (Jones et al., 2015) than the CC system. While the negative impacts of HS on the productivity and wellbeing of laying chickens are well documented, the mechanistic basis underlying such outcomes is still poorly understood. Specifically, very few studies have directly corelated the physiological changes and transcriptomic responses with the observed reduction in egg production and quality during HS conditions (Kim et al., 2024; Habashy et al., 2025). Additionally, most of the studies to date have been conducted using CC systems, however, as the industry is shifting toward the CF system, it is essential to characterize how HS impacts laying chickens under these newer housing conditions. In this study, we investigated the effects of chronic HS on commercial layers housed in a CF system, with particular emphasis on egg production, egg quality, feed intake, blood physiological parameters and selected gene expressions. Importantly, we have corelated the physiological changes in blood parameters and transcriptomic alterations of key genes in uterus (shell gland) directly involved in egg formation to determine how HS-mediated molecular alterations contribute to the observed production losses. The findings of this study will help identify nutritional and managemental interventions aimed at correcting the observed biochemical and molecular alterations, thereby mitigating the adverse effects of chronic HS in commercial laying chickens.

## 2 Materials and methods

### 2.1 Birds and experimental design

**FIGURE 1:**
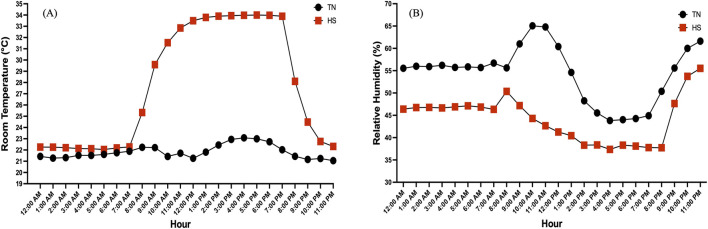
Hourly **(A)** room temperature (°C) and **(B)** relative humidity (RH%) recorded under thermoneutral (TN) and heat stress (HS) conditions across a 24-h period.

Two hundred and forty Hy-Line W-36 layers were raised on floor pens from day of hatch to 25th weeks of age under feeding, lighting, and temperature conditions recommended by the Hy-Line management guidelines. At 26th week of age, birds were randomly allocated to two separate rooms, with two temperature treatments: thermoneutral (TN, temperature set to 22 °C) and cyclic heat stress (HS, temperature set to 35 °C for 12 h followed by 22 °C for 12 h, from 7 a.m. to 7 p.m.). Under our experiment conditions, the HS room took approximately 4 h to reach the target temperature, 35 °C (Figure 1). The experiment was conducted over an 8-week period (26–33 weeks of age), corresponding to the peak egg production phase of the flock. Each room/treatment housed 120 hens divided in 6 pens (20 hens/pen), at a stocking density of 0.139 m^2^/hen. Therefore, pen was the experimental unit in this study, and all statistical analyses were conducted using pen-level averages. Feed (Table 1) and water were provided *ad libitum* and the birds were maintained under a 16-h light and 8-h dark cycle throughout the 8-week duration of the experiment. All experimental procedures conducted in this study were approved by Institutional Animal Care and Use Committee of the Ohio State University (2024A00000066).

**TABLE 1:** Item description and analysis (%) of formulated layer feed.

Items	Percentages (%)
Ground corn	62.35
Soybean meal (47% crude protein)	18.3
Corn gluten meal (60% crude protein)	4.9
Animal fat	3.5
Limestone	9.3
Dicalcium phosphate (18.5% P)	0.85
Plain salt (sodium chloride)	0.4
DL-methionine (99%)	0.25
Vitamin-mineral premix	0.15
Feed analysis	
Crude protein, minimum	14.004
Lysine, minimum	0.685
Methionine, minimum	0.455
Crude fat, minimum	6.411
Crude fiber, minimum	2.338
Calcium, minimum	3.387
Calcium, maximum	4.387
Phosphorous, minimum	0.456
Salt, minimum	0.398
Salt, maximum	0.898
Selenium (added), minimum	0.3 ppm

### 2.2 Room temperature and relative humidity measurement

The air temperature and relative humidity (RH%) in the TN and HS tests rooms were continuously measured at sampling rate of 5 min for the duration of the study period using HOBO sensors (HOBO U23 Pro v2, onsetcomp.com). The HOBO air temperature and relative humidity sensors were installed at the center of two pens in each of the test room with a height of about 2.5 ft. to measure the average thermal conditions in the test rooms. The 5-min measurements within each hour were averaged to obtain an hourly mean value. To depict the cyclic daily pattern in temperature and RH% between the TN and HS rooms, one representative day from the experiment period was selected for graphical presentation.

### 2.3 Layer performance parameters

Body weight (BW) was measured weekly from the five randomly selected birds per pen. Similarly, feed intake (FI) per pen and body temperature from the randomly selected birds were measured weekly, whereas egg production and egg weight were recorded daily in each of the pens. Body temperature was measured by inserting the probe of a digital thermometer into the cloaca. Mortality and breathing behavior were recorded every day. Hen-day egg production (HDEP%) was calculated by dividing the total number of eggs produced each day per pen by the number of hens in that pen on the corresponding day. Average egg weight per pen was determined and the total egg mass was then reported for each treatment. Feed conversion ratio (FCR) was calculated as the ratio of FI to egg mass.

### 2.4 Egg quality parameters

Egg quality parameters were measured after 6- and 8-weeks of HS for each of the treatments with five eggs per pen (30 eggs/treatment). Texture analyzer instrument (Stable Micro Systems, Godalming, UK) was used to measure the individual egg weight, shell breaking strength, shell deformation, shell thickness, shell weight, egg albumen height, Haugh unit, vitelline membrane strength, yolk color, yolk height, yolk width, and yolk index. Shell weight (g) was measured along with the shell membrane after removing all albumen and egg content. Yolk color was evaluated using the DSM yolk color fan, with scores ranging from 1 to 15, where the higher values indicate a darker yolk. Yolk index was calculated as the ratio of yolk height (mm) and yolk width (mm) and is used to assess the shape change in the yolk during storage.

### 2.5 Blood physiological parameters

At the end of study, blood samples were collected from the wing vein of twelve birds per treatment (2 birds/pen) in heparinized tube. Immediately, 100 µL blood was loaded into iSTAT CG8+ cartridge and analyzed using an i-STAT Alinity v Blood Analyzer (Zoetis, United States). The CG8+ cartridge quantifies the levels of thirteen blood parameters, including pH, partial pressure of carbon dioxide (PCO_2_) and oxygen (PO_2_), total carbon dioxide (TCO_2_), bicarbonate (HCO_3_), base excess (BE), oxygen saturation (sO_2_), sodium (Na^+^), potassium (K^+^), ionized calcium (iCa^2+^), glucose (Glu), hematocrit (Hct) and hemoglobin (Hb).

### 2.6 RNA extraction and gene expression

At the last day of study, six birds from each treatment (1 bird/pen) were randomly selected, euthanized through CO_2_ asphyxiation at 45% flow rate of chamber/minute, and shell gland was collected in RNA later and stored in −20 °C until further analysis. RNA was isolated from the shell gland using the Monarch® Total RNA Miniprep Kit (New England Biolabs®, Ipswich, MA, United States) following manufacturer’s instructions. The concentration of RNA was then adjusted to 800 ng and subsequently reverse transcribed to cDNA using the LunaScript® RT SuperMix Kit (New England Biolabs®, United States) and a gradient thermocycler (Bio-Rad Laboratories, Hercules, CA, United States). The thermocycler was programmed to operate at 25 °C for 2 min, 55 °C for 10 min, and 95 °C for 1 min. Quantitative real-time (RT) PCR was then conducted on a Bio-Rad CFX Connect Real-Time System (Bio-Rad Laboratories, Hercules, CA, United States). Each RT-PCR reaction mixture (20 μL total volume) consisted of 10 μL of Luna® Universal qPCR Master Mix (New England Biolabs®, United States), 0.5 μL each of forward and reverse primers (10 μM), 2 μL of cDNA (10 ng/μL), and 7 μL of nuclease free water. The qPCR was performed with an initial denaturation at 95 °C for 1 min, followed by 40 cycles of denaturation (95 °C for 15 s) and extension (60 °C for 30 s). A final melt curve analysis was conducted by gradually increasing the temperature from 60 °C to 95 °C in 0.5 °C increments, with fluorescence recorded at each step. We measured the mRNA expression of various genes associated with eggshell formation and mineralization, including the calbindin 1 (*CALB1*, a calcium-binding protein), IP3 receptor-3 (*ITPR3*, an intracellular calcium release channel), solute carrier family 4 member 4 (*SLC4A4*, a Na^+^/HCO_3_^−^ co-transporter), solute carrier family 4 member 9 (*SLC4A9*, a Cl^−^/HCO_3_^−^ exchanger), and osteopontin (*OPN*). The list of genes utilized in this study, their accession IDs, sequences and product size are summarized in Table 2. The cycle threshold (Ct) value for each gene was normalized to the internal control gene, *YWHAZ* (Hassanpour et al., 2019) and the relative fold change was calculated using the 2^−ΔΔCt^ method (Livak and Schmittgen, 2001).

**TABLE 2:** List of genes, their accession number, primer sequences and product size used for quantitative real-time PCR.

Gene symbol	Accession ID	Primer sequence (5′-3′)	Product size (bp)
*CALB1*	NM_205513.1	F: TCTGGCACCACTACGACTCC	148
		R: GCCTTGCCATACTGGTCCAC	
*ITPR3*	XM_015298987.1	F: CCGAGGTGGAGACCTTTGTC	75
		R: ACAGGTCCGAGAGGTAGTCC	
*SLC4A4*	XM_015276405.1	F: CGGCACCAGACCAAGAAGTC	65
		R: GCACTGGAGACGGTCTTTCC	
*SLC4A9*	XM_015293554.1	F: CGAGGACTACGGTCTGGACT	109
		R: GTACTGCACCAGGTGACTCG	
*OPN*	NM_204535.4	F: AAGAGGCCGTGGATGATGATG	254
		R: ATCCTCAATGAGCTTCCTGGC	
*YWHAZ*	NM_001031343.1	F: AGGAGCCGAGCTGTCCAATG	84
		R: CTCCAAGATGACCTACGGGCTC	

*CALB1*, calbindin 1; *ITPR3*, IP3 receptor3; *SLC4A4*, solute carrier family 4 member 4; *SLC4A9*, solute carrier family 4 member 9; *OPN*, osteopontin; *YWHAZ*, tyrosine 3-monooxygenase/tryptophan 5-monooxygenase activation protein zeta; F, forward; R, reverse.

### 2.7 Statistical analysis

Data from production parameters and egg quality parameters were analyzed using the following statistical model:

$$y_{ij}=\mu +\alpha_{i}+\beta_{j\left(i\right)}+\varepsilon_{ij}$$

where y_ij_ represents the response variable measured from the *j* ^th^ pen within the *i* ^th^ temperature treatment; μ is the overall mean; $\alpha_{i}$ is the fixed effect of temperature, where i=1,2 (TN and HS); $\beta_{j\left(i\right)}$ is the random effect of pen *j* nested within temperature treatment *i*; and $\varepsilon_{ij\;}$ is the residual error term. These data were analyzed using a mixed-model approach implemented in SAS® OnDemand for Academics (SAS Institute Inc., 2024) using the PROC MIXED procedure. For the blood biochemical parameters and relative mRNA expression levels of shell gland genes, data were analyzed using Student’s *t*-test. A p-value less than 0.05 was considered statistically significant.

## 3 Results

### 3.1 Room temperature and relative humidity

Figures 1A,B represent the hourly room temperature and RH% recorded under TN and HS conditions across a 24-h period. The TN room maintained at stable average temperature of approximately 21.8 °C, while the HS room exhibited a cyclic temperature pattern, rising from ∼22 °C in the early morning to a peak of ∼34 °C during the daytime, followed by a gradual decline. As temperature increased, RH% decreased during the hotter hours and subsequently increased as the temperature dropped.

### 3.2 Body weight, body temperature, and mortality

**FIGURE 2:**
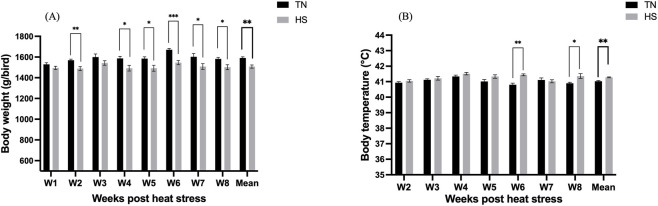
Effect of chronic heat stress on **(A)** body weight (g/bird), and **(B)** body temperature (°C) in laying hens over the study period. Data were analyzed using the linear mixed-effect model with pen as a random effect. *p < 0.05, **p < 0.01, ***p < 0.001.

Exposure to chronic HS significantly (p < 0.05) reduced BW by week 2 and this reduction persisted through the end of the study compared to the TN birds, with average overall weights of 1508.5 and 1590.7 g/bird, respectively (Figure 2A). Body temperature varied between the groups, particularly at 6- and 8-weeks post-HS, in which the HS birds had significantly higher (p < 0.05) body temperature than the TN birds (Figure 2B). There was no mortality recorded throughout the study period, although the HS birds exhibited clear signs of HS, including open-mouth breathing (panting).

### 3.3 Feed intake and feed conversion ratio

**FIGURE 3:**
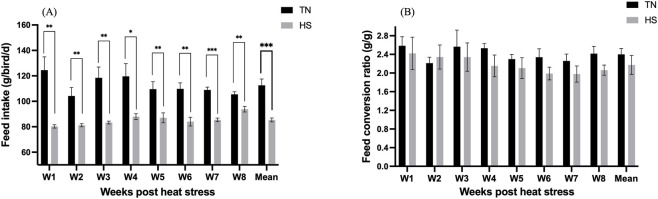
Effect of chronic heat stress on **(A)** feed intake (g of feed consumed/hen/d), and **(B)** feed conversion ratio (FCR) (g of feed consumed/g of egg mass) in laying hens over the study period. Data were analyzed using the linear mixed-effect model with pen as a random effect. *p < 0.05, **p < 0.01, ***p < 0.001.

The HS hens consumed significantly (p < 0.01) less feed than TN group throughout the study period (Figure 3A), with the mean FI of 85.4 and 112.57 g of feed/bird/day, respectively. However, the FCR did not differ significantly between the treatment groups for the entire study timeline (Figure 3B).

### 3.4 Hen day egg production

**FIGURE 4:**
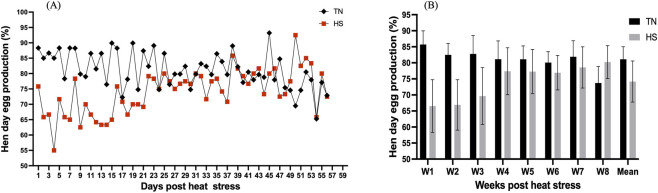
Effect of chronic heat stress on **(A)** daily hen day egg production (HDEP%), and **(B)** weekly HDEP% in laying hens across the study period. Data were analyzed using the linear mixed-effect model with pen as a random effect.

Exposure to HS caused an initial decline in daily HDEP% during the first 3 weeks of the experiment, followed by gradual recovery and stabilization thereafter (Figure 4A). However, when production data were summarized on a weekly basis, no statistically significant differences (p > 0.05) were detected between the TN and HS groups throughout the experimental period (Figure 4B).

### 3.5 Egg weight and quality

**FIGURE 5:**
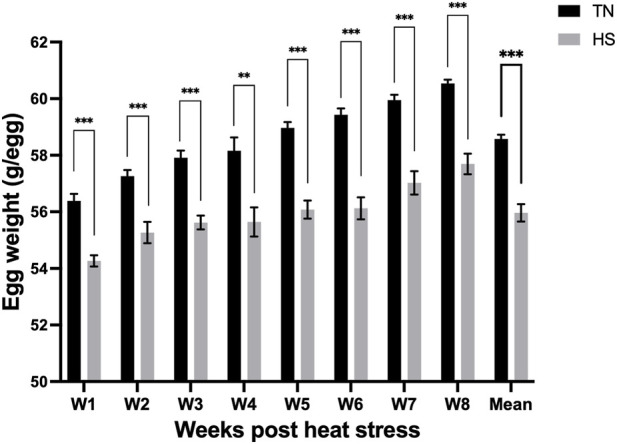
Effect of chronic heat stress on egg weight (g/egg) across the study period. Data were analyzed using the linear mixed-effect model with pen as a random effect. **p < 0.01, ***p < 0.001.

The HS hens laid significantly (p < 0.001) lighter eggs—by nearly 3 g—compared to TN hens, and the trend persisted for the entire study period (Figure 5). Eggs from the HS hens showed significant decline in shell weight and shell breaking strength at 6- and 8-weeks of HS, however had less incidence of shell deformation (Table 3). At week 6 of HS, TN eggs had a darker yellow yolk compared to HS eggs, however, this pattern reversed by week 8, with HS eggs exhibiting darker yolks. Moreover, HS eggs had significantly shorter yolk widths than TN eggs, with the difference being most pronounced at week 8. No significant differences were observed in other egg quality parameters.

**TABLE 3:** Effect of chronic heat stress on various egg quality parameters in laying chickens.

Treatment	Egg weight (gm)	Shell weight (g)	Shell thickness (mm)	Shell breaking strength (N)	Shell deformation (mm)	Egg albumen height (mm)	Haugh unit	Vitelline membrane strength (N)	Yolk color (1–15)	Yolk height (mm)	Yolk width (mm)	Yolk index
6 weeks post-HS												
TN	59.68	6.83	0.385	66.3^a^	0.798^a^	14.63	113.73	0.118	9.33^a^	14.87	40.09	0.363
HS	55.7	6.43	0.372	57.74^b^	0.701^b^	13.66	109.55	0.119	8.57^b^	14.07	39.95	0.354
SEM	1.39	0.145	0.037	1.55	0.028	1.8	6.31	0.003	0.162	0.725	0.13	0.007
p-value	0.0685	0.0891	0.81	0.0039	0.03	0.71	0.65	0.9694	0.0062	0.546	0.4557	0.423
8 weeks post-HS												
TN	60.91^a^	7.53^a^	0.474	65.25^a^	0.805	11.31	100.15	0.134	9.46^b^	14.26	40.83^a^	0.348
HS	56.64^b^	6.61^b^	0.419	58.21^b^	0.746	11.8	100.14	0.119	9.81^a^	13.5	39.65^b^	0.341
SEM	0.81	0.166	0.035	2.22	0.025	0.818	3.69	0.006	0.08	0.247	0.306	0.007
p-value	0.0049	0.003	0.3076	0.0499	0.1237	0.6744	0.998	0.128	0.0121	0.054	0.0209	0.5

TN, thermoneutral (22 °C); HS, heat stress (35 °C for 12 h followed by 22 °C for 12 h); SEM, standard error of mean. Superscripts a, b are used to indicate significant parameters (p < 0.05) across columns.

### 3.6 Blood biochemical parameters

Exposure to chronic HS significantly lowered blood pH (*p* < 0.05), while increasing PCO_2_ and iCa levels compared to TN groups (Table 4). No significant differences were observed between HS and TN groups for PO_2_, HCO_3_^−^, BE, sO_2_, TCO_2_, Na^+^, K^+^, Glc, hematocrit, or hemoglobin levels.

**TABLE 4:** Effect of chronic heat stress on blood biochemical parameters in laying chickens.

Treatment	pH	PCO_2_ (mmHg)	PO_2_ (mmHg)	HCO_3_ (mE q/L)	BE, ecf (mE q/L)	sO_2_ (%)	TCO_2_ (mE q/L)	Na (mE q/L)	K (mE q/L)	iCa (mmol/L)	Glu (mg/dL)	Hct (%PCV)	Hb (g/dL)
TN	7.351^a^	38.79^b^	62.4	20.49	−4.3	83.8	21.6	147	4.68	1.47^b^	231	23.8	8.09
HS	7.267^b^	48.21^a^	68	20.9	−5.2	84.11	22.2	146.3	4.71	1.61^a^	230	23.6	8.04
SEM	0.017	2.57	2.11	0.651	0.59	1.21	0.731	0.68	0.098	0.033	3.12	0.613	0.21
p-value	0.0034	0.0169	0.0713	0.6537	0.2743	0.855	0.5456	0.4252	0.8215	0.0081	0.7665	0.8766	0.878

TN, thermoneutral (22 °C); HS, heat stress (35 °C for 12 h followed by 22 °C for 12 h); PCO_2_, partial pressure of carbon dioxide; PO_2_, partial pressure of oxygen; HCO_3_, bicarbonate; BE, ecf, base excess in extracellular fluid; sO_2_, oxygen saturation; TCO_2_, total carbon dioxide; Na, sodium; K, potassium; iCa, ionized calcium; Glu, glucose; Hct, hematocrit; Hb, hemoglobin; SEM, standard error of the mean. Superscripts.a, b are used to indicate significant parameters (p < 0.05) across columns.

### 3.7 Gene expression

**FIGURE 6:**
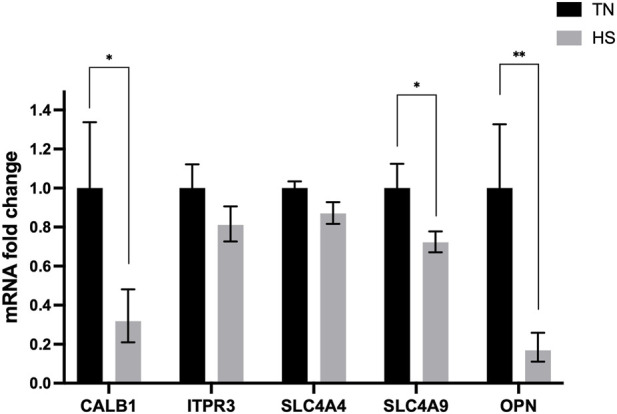
Effect of chronic heat stress on mRNA expression of genes related with eggshell formation and mineralization in shell gland. Data was analyzed using the Student’s t-test. *CALB1*, calbindin 28K; *ITPR3*, IP3 receptor3; *SLC4A4*, solute carrier family 4 member 4; *SLC4A9*, solute carrier family 4 member 9; *OPN*, osteopontin. *p < 0.05; **p < 0.01.

Chronic HS downregulated the expression of genes associated with eggshell formation and mineralization in eggshell gland (Figure 6). The relative mRNA expression of *CALB1*, *SLC4A9* and *OPN* were significantly downregulated (p < 0.05) in the HS birds compared with TN birds. However, the expression of the *ITPR3* and *SLC4A4* remained similar (p > 0.05) between groups.

## 4 Discussion

This study was conducted to evaluate the effects of chronic HS on growth performance, egg production, egg quality, blood biochemical indices, and the expression of selected genes in shell glands in Hy-Line W-36 birds housed in a CF system. Our findings revealed that HS negatively influenced body weight, feed intake, early egg production, egg weight, and shell quality. In addition, HS altered blood acid–base balance and iCa levels as well as downregulated key shell gland genes involved in mineralization. These findings provide further insight into how prolonged HS compromises the productivity and physiology of commercial layers, particularly under CF system.

In the present study, chronic HS significantly reduced BW in laying hens compared to TN controls. This reduction can be attributed to the combined effects of decreased feed intake and the additional energy expenditure required for thermoregulation. Under thermal stress, birds redirect available energy toward dissipating excess heat rather than growth (Papanikolaou et al., 2025), resulting in a negative energy balance. Previous studies have reported that HS leads to the mobilization of body reserves and breakdown of muscle protein to meet production demands (Furukawa et al., 2016; Ma et al., 2021), which aligns with the reduction in BW observed in our trial. Although BW tended to be lower in HS birds at weeks 1 and 3, no statistically significant differences were observed compared to TN birds. In contrast, FI in HS birds remained significantly lower throughout the study period. Despite BW and FI are generally corelated, reductions in FI might not always perfectly translate into proportional BW loss as it can also be affected by residual feed efficiency traits and metabolic needs (Willems et al., 2013; Lewis and Emmans, 2020).

The significant reduction of FI in HS birds compared to TN birds might be related with the anorexic effects of heat exposure. This suppression of appetite has been linked to elevated secretion of leptin and adiponectin, as well as the expression of their receptors. Leptin, in turn, activates the hypothalamic axis and suppresses feed intake (Bernabucci et al., 2009; Morera et al., 2012). Despite this reduction of FI observed in the HS group, the FCR remained comparable with the TN group in our study, which aligns with reports from previous findings (Bozkurt et al., 2012; Kim et al., 2020). However, there are lots of inconsistent findings regarding the effect of HS on feed efficiency. Some studies have demonstrated a worsened FCR under HS conditions (He et al., 2018), whereas other studies have observed an improved FCR in HS birds (Barrett et al., 2019; Kim et al., 2024) which has been linked with the mobilization of body reserves to sustain egg production (Song et al., 2012). These discrepancies in FCR are likely the results of different experimental conditions, including the duration and severity of heat exposure, as well as differences in bird age and housing system.

The decrease in egg weights observed in HS chickens compared to controls in our study is consistent with previous findings reporting a similar trend of egg weight reduction under HS conditions (Mashaly et al., 2004; Allahverdi et al., 2013). This reduction in total egg weight has been linked with the reduction in yolk weight (Chen et al., 2023) and albumen weight (Barrett et al., 2019). In this study, HS birds exhibited an initial decline in HDEP% during the first 3 weeks, which then recovered by fourth week. Previous studies have also reported that continuous exposure to HS severely decreases HDEP% of layer chickens (Rozenboim et al., 2007; Kim et al., 2024). In another study, only continuous HS significantly reduced HDEP%, while cyclic HS (8 h a day) did not significantly affect the HDEP% compared to controls (Mashaly et al., 2004). Together, these studies suggest that the duration of heat exposure plays a critical role in determining its effects on laying performance.

The detrimental effects of HS on eggshell quality are well documented, and have been associated with acid-base imbalance, impaired nutrient absorption, and altered calcium metabolism (Mahmoud et al., 1996; Bahadoran et al., 2018). In this study, eggs from HS birds exhibited significantly lower shell breaking strength and shell weight, which potentially could increase the egg loss during handling and transportation. Together, these results confirm that prolonged HS compromises eggshell integrity, in line with earlier reports of decreased shell strength and thickness (Kim et al., 2020; Kim et al., 2022).

**FIGURE 7:**
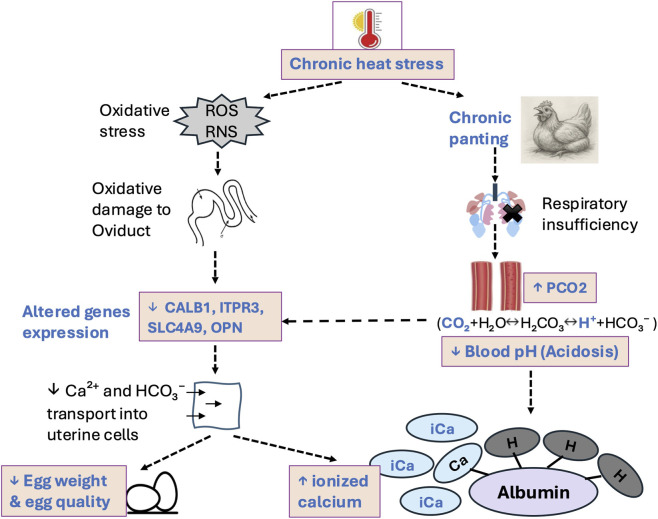
Proposed mechanisms underlying the adverse effects of chronic heat stress in commercial laying hens. These include alteration in blood biochemical parameters and oxidative stress-mediated changes in genes expression within the shell gland, ultimately resulting in observed production losses. PCO_2_, partial pressure of carbon dioxide; H^+^, proton ion; ROS, reactive oxygen species; RNS, reactive nitrogen species; *CALB1*, calbindin 28K; *ITPR3*, IP3 receptor3; *SLC4A9*, solute carrier family 4 member 9; *OPN*, osteopontin.

Blood biochemical parameters may provide valuable insights into the physiological condition associated with the egg mass and eggshell formation. Previous research has demonstrated that HS disrupts calcium metabolism and decreases calcium concentration in blood plasma, leading to thinner eggshells (El-Tarabany, 2016; Cruvinel et al., 2021). Herein, we observed reduced blood pH in HS birds that could be the result of multiple physiological disturbances, including hyperventilation-induced respiratory alkalosis followed by metabolic compensation, impaired renal function reducing acid-base regulation, and increased lactic acid accumulation due to tissue hypoxia (Aryal et al., 2025). However, the PCO_2_ level was increased in HS birds despite the expectation of excessive CO_2_ loss due to panting. This paradox may indicate a shift from respiratory to metabolic disturbances during prolonged HS. Initially, panting reduces PCO_2_, leading to respiratory alkalosis, but over time, fatigue of respiratory muscles, reduced ventilation efficiency, or impaired gas exchange due to pulmonary damage may hinder CO_2_ elimination (Brugaletta et al., 2022; Wu et al., 2023). Furthermore, increased metabolic rate and potential tissue hypoxia under HS may contribute to elevated endogenous CO_2_ production, outweighing respiratory losses and ultimately resulting in higher circulating PCO_2_ concentrations (de Souza et al., 2016; Lian et al., 2021). In addition, the increased level of iCa observed in HS birds may be attributed to the reduction in blood pH in those birds. There is an established inverse relationship between Ca^2+^ levels and blood pH (Axelrod, 1961; McCudden, 2013). As blood pH decreases (acidosis), proton ion (H^+^) competes with Ca^2+^ for binding sites on plasma proteins, particularly albumin, resulting in a greater proportion of calcium remaining in its free, ionized form (Figure 7).

HS condition induces excessive ROS production, resulting in oxidative damage to proteins, lipids, carbohydrates, and DNA (Emami et al., 2020). One possible mechanism by which HS results in oxidative stress in internal organs, including the intestinal and reproductive tract, is the redistribution of blood flow towards the external surface (skin) of the body to promote heat dissipation. This compensatory response reduces blood flow to internal organs, resulting in hypoxic condition and subsequent development of oxidative and nitrosative stress (Rostagno, 2020). Moreover, study from Li et al. (2020) suggested that HS-induced elevation in corticosterone likely increases oxidative stress, that in turn activated the FasL/Fas and TNF-α apoptotic pathways in follicular cells, ultimately impairing follicular development and egg production. Similarly, separate study has demonstrated that oxidative stress reduced the expression of genes associated with cell survival and steroidogenic function, including *SIRT1*, while increasing the expression of pro-apoptotic regulators *P53* and *FoxO1* in chicken ovary, thereby reducing ovarian function and laying performance (Wang et al., 2021). In the present study, the observed downregulation of genes associated with uterine ion transport and biomineralization (*CALB1, SLC4A9,* and *OPN)* may similarly reflect oxidative injury and subsequent apoptosis occurring within uterine tissues under HS condition. Consistent with this, a previous study reported that HS downregulated key uterine ion transporter genes (*ITPR3*, *SLC4A4*, and *SLC4A9*), impairing uptake of Ca^2+^ and HCO_3_^−^ into uterine epithelial cells (Bahadoran et al., 2018). This disruption may explain the elevated circulating iCa observed in our study, alongside reduced egg weight and weakened shell strength. Consistently, reductions in eggshell quality have also been linked to decreased *CALB1* localization in eggshell gland and intestine (Ebeid et al., 2012) and to altered serum electrolytes levels during HS (Kim et al., 2024).

In conclusion, chronic HS exhibited detrimental effects in layer chickens housed in CF system. These adverse effects include reduced BW, feed consumption, initial egg production rate, egg weight, and shell breaking strength. These changes were accompanied by acid–base imbalance and altered calcium metabolism, reflected as lower blood pH and elevated PCO_2_ and iCa levels. Future studies are warranted to delineate the mechanism of HS-induced oxidative injury in chicken reproductive tract and underlying alterations in production performance, immune responses, disease susceptibility and gut microbiota diversity in HS chickens raised in CF system. Moreover, development and field-scale evaluation of effective nutritional supplements and physical cooling methods in commercial settings will be critical for improving resilience to HS in commercial laying operations.

## Data Availability

The raw data supporting the conclusions of this article will be made available by the authors, without undue reservation.
